# Refractory Pruritus in Advanced Liver Disease: Exploring the Overlooked Role of Basic Emollient Care

**DOI:** 10.7759/cureus.117839

**Published:** 2026-10-13

**Authors:** Sumiyya Rashid, Simranjot Kaur, Amal Qaisar, Danyaal Anzar

**Affiliations:** 1 Palliative Care, University Hospitals of Leicester NHS Trust, Leicester, GBR; 2 Accident and Emergency, University Hospitals of Leicester NHS Trust, Leicester, GBR; 3 Nephrology, University Hospitals of Leicester NHS Trust, Leicester, GBR; 4 Hematology, Imperial College Healthcare NHS Trust, London, GBR

**Keywords:** alcohol-related liver disease, decompensated cirrhosis, dermatology, emollients, end-of-life care, hepatic pruritus, palliative care, symptom control

## Abstract

Pruritus is a prevalent yet frequently under-recognized symptom of chronic liver disease. Symptom control becomes particularly challenging in palliative care, where treatment is limited by frailty, comorbidity, and a shift in focus from cure to comfort. We present a 70-year-old woman with decompensated alcohol-related liver disease and long-standing chronic pruritus who was admitted following a fall and subsequently transitioned to palliative care. Her pruritus markedly worsened during admission and improved partially with antihistamines and levomepromazine. Liver disease was considered the leading cause. Opioid therapy, dehydration, and disruption of her usual skin-care routine were considered contributing factors. Collateral history established that her pruritus had previously been managed at her care home with regular emollients, a regimen that was applied less consistently following admission. Dermatological assessment found no primary skin condition. The most noticeable improvement followed the reinstatement of intensive emollient therapy, although her symptoms fluctuated and never fully resolved. This case suggests that treatment-refractory pruritus at the end of life may sometimes reflect a lapse in basic supportive care rather than disease progression alone. It also highlights the value of collateral history and combined palliative and dermatological assessment in managing these patients.

## Introduction

Pruritus is the sensation of itch that provokes an urge to scratch [1]. It is a common presenting symptom in dermatology but also frequently accompanies systemic disease [2]. In patients receiving palliative care, pruritus can be a significant source of distress at the end of life, affecting comfort, sleep, and quality of life [2]. However, unlike pain, nausea, and agitation, it does not always receive the same systematic assessment or escalation of treatment [3].

Pruritus is broadly classified according to its underlying mechanism: dermatological (arising from primary skin disease), systemic (secondary to conditions such as liver disease, renal failure, or malignancy), neuropathic, psychogenic, and mixed, in which several of these mechanisms coexist [1]. In chronic liver disease specifically, pruritus is most strongly associated with cholestatic conditions such as primary biliary cholangitis and primary sclerosing cholangitis [4,5]. Its pathophysiology is thought to involve retained bile acids, endogenous opioids, and more recently identified mediators such as lysophosphatidic acid, although no single mechanism fully explains the symptom [4,5]. Pruritus is less strongly associated with noncholestatic liver disease, including alcohol-related liver disease (ARLD), so its presence in such patients presents a more ambiguous clinical picture. This unpredictability, combined with the absence of a single underlying mechanism, makes pruritus difficult to manage in practice, particularly in palliative and end-of-life care.

Basic emollient therapy has been shown to reduce pruritus severity in xerosis-associated conditions and remains a mainstay of initial treatment for itch associated with dry skin, regardless of its underlying cause [6]. When pruritus becomes severe or refractory, there is a tendency to escalate to systemic or sedative therapy. This risks overshadowing simpler, evidence-based measures.

We present a patient with decompensated ARLD who developed severe, treatment-refractory pruritus during an inpatient admission and subsequent transition to palliative care. This case illustrates the value of a multidisciplinary team approach to patient care and highlights the risk of attributing refractory itch to disease progression alone without first reassessing simpler, potentially reversible skin-directed contributors.

## Case presentation

A 70-year-old woman with ARLD complicated by recurrent tense ascites (previously requiring outpatient drainage) and hepatic encephalopathy was admitted after an unwitnessed fall at her care home. Her medical history included atrial fibrillation, colon cancer treated with a hemicolectomy, with no evidence of recurrence, hypertension, glaucoma, and osteoarthritis. She had an existing Recommended Summary Plan for Emergency Care and Treatment (ReSPECT) form documenting do-not-attempt-cardiopulmonary-resuscitation (DNACPR) status and a comfort-focused ceiling of care.

On admission, imaging confirmed a comminuted right distal radius fracture with ulnar styloid involvement and acute right 10th and 11th rib fractures sustained during the fall. Her wrist fracture was managed conservatively with manipulation and casting. CT of the head was unremarkable. CT of the abdomen and pelvis confirmed decompensated liver disease with ascites and portal hypertension (Figure 1). She also developed stage 1 acute kidney injury and delirium; the latter was considered multifactorial, with pain, constipation, infection, and hepatic encephalopathy all contributing.

**Figure 1:**
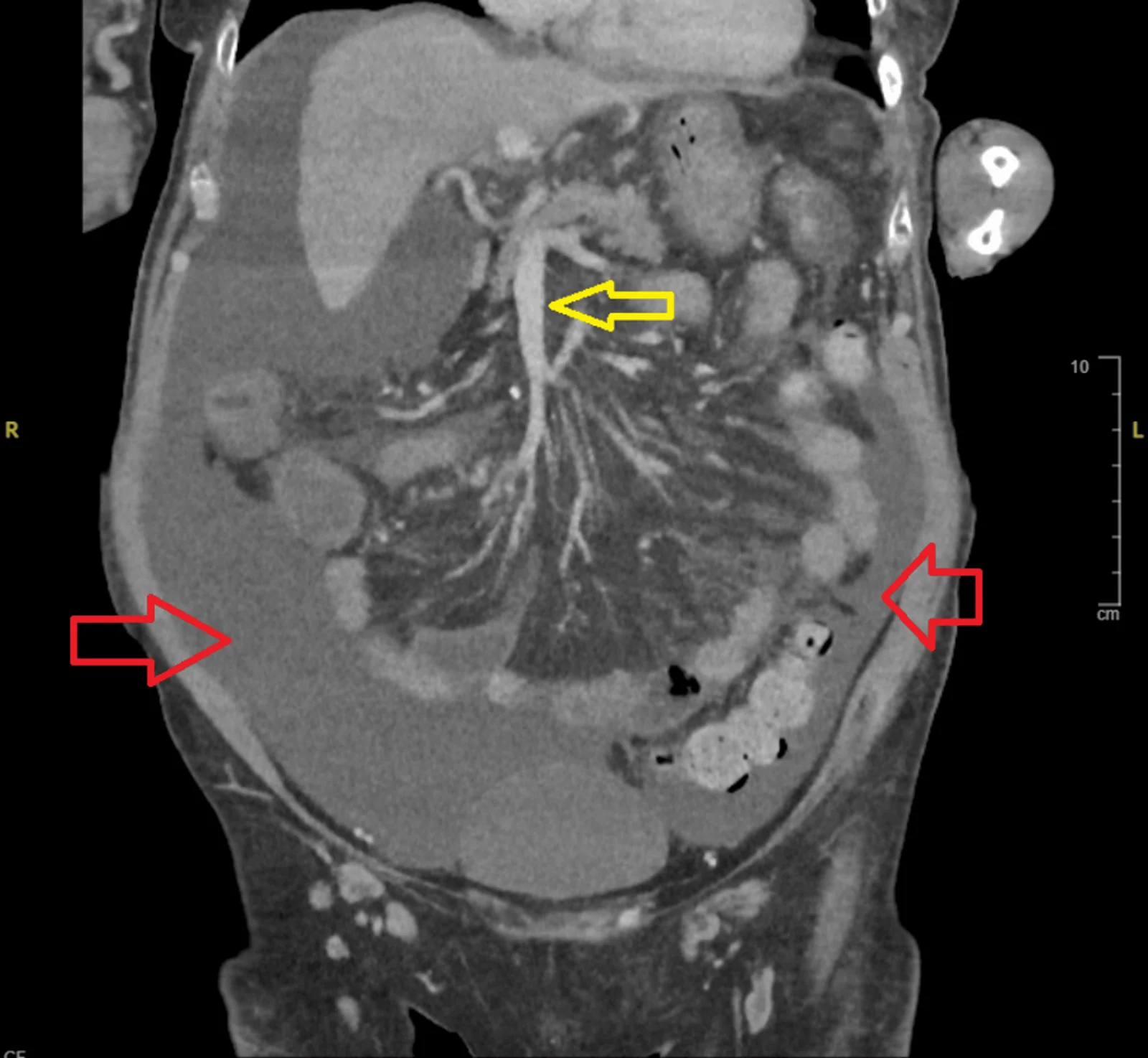
CT of the abdomen and pelvis in a patient with decompensated liver disease Coronal contrast-enhanced CT showing a large-volume fluid collection surrounding centrally displaced bowel loops, consistent with ascites (red arrows). An enlarged superior mesenteric vein is also visible near the porta hepatis (yellow arrow), consistent with portal hypertension.

Gastroenterology managed her decompensated liver disease with lactulose, rifaximin, spironolactone, and vitamin K. Liver biochemistry on admission is summarized in Table 1 (Child-Pugh class C; Model for End-Stage Liver Disease score of 19). An ascitic tap excluded spontaneous bacterial peritonitis. Given her history of recurrent ascites, a long-term indwelling ascitic drain was inserted on day 16 of admission. Ascites was drained to dryness with albumin cover (100 mL of 20% human albumin solution per 3 L drained).

**Table 1:** Liver biochemistry on admission

Parameter	Result	Reference range	Unit
Bilirubin	37	3-17	µmol/L
Alanine aminotransferase (ALT)	33	7-56	U/L
Alkaline phosphatase (ALP)	183	30-130	U/L
Gamma-glutamyl transferase (GGT)	Not measured	5-55 (female)	U/L
Albumin	28	35-50	g/L
International normalized ratio (INR)	2.1	0.8-1.2	-

Onset and evolution of pruritus

Within days of drain insertion, she developed increasing abdominal pain alongside worsening pruritus, initially affecting her limbs and trunk. The drain site was erythematous, and her symptoms were initially attributed to dehydration and a drain-site infection. She was treated with intravenous fluids, antibiotics, and albumin, and the drain was removed. Over the following week, the itch became widespread and a persistent source of distress, with extensive excoriations and skin breakdown (Figure 2). In addition to regular emollients, fexofenadine was started empirically but provided only partial relief.

**Figure 2:**
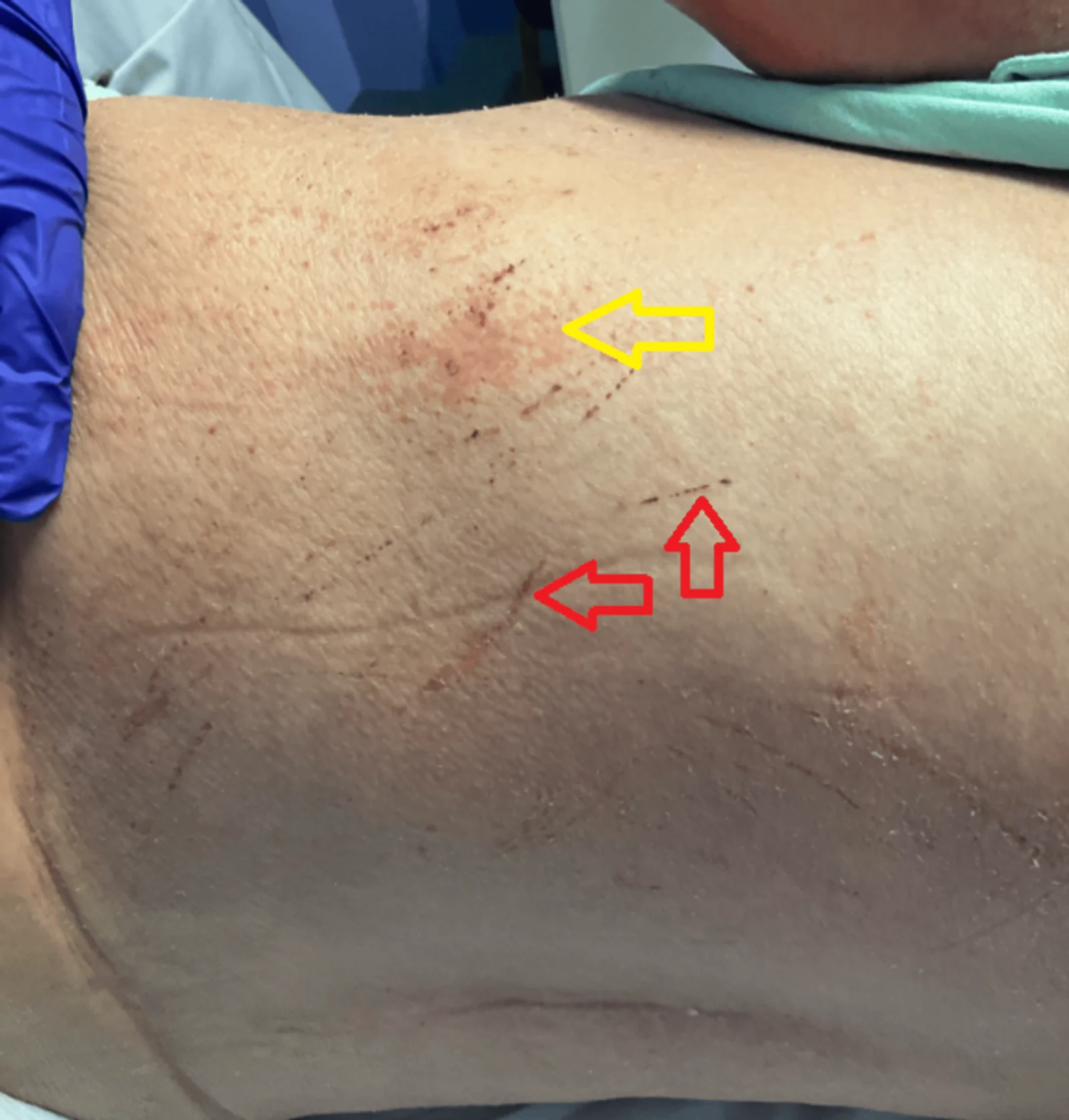
Extensive excoriations on the posterior trunk secondary to chronic pruritus Clinical photograph demonstrating widespread linear excoriations (red arrow) and areas of superficial skin breakdown (yellow arrow), consistent with persistent scratching in the context of severe pruritus. No primary inflammatory dermatosis was evident.

Collateral history from her next of kin (NOK) indicated that the pruritus had been present for around a year before admission, although the dermatology team had previously declined to review her because the itch was attributed to her liver disease. Her family felt that her symptoms had worsened in hospital, attributing this to less frequent emollient application and dry skin following large-volume ascitic drainage; neither explanation was confirmed at the time. The pruritus persisted, and a palliative care referral was made. The palliative care team recognized an overall deterioration in her clinical condition, estimated her prognosis at weeks to months, and commenced a continuous subcutaneous infusion (CSCI) of morphine and midazolam for symptom control.

As the pruritus remained severe and refractory, the palliative care team reviewed the differential diagnoses. Repeat liver biochemistry around the time her pruritus was most severe showed similar findings (Table 2), without evidence of a worsening cholestatic picture. Uremic pruritus was also considered but deemed unlikely (estimated glomerular filtration rate (eGFR) decreased from 90 to 49 mL/min/1.73 m², and urea increased from 8.8 to 16.6 mmol/L). Chronic liver disease and dry skin were considered the most likely causes. Levomepromazine was added to the CSCI to help control agitation and itch. The dermatology team reviewed her and found no active inflammatory dermatosis. Xerosis and severe excoriations attributable to constant itching were noted on examination. Management included mometasone, menthol-containing emollients, fexofenadine at 180 mg once daily, and a review of morphine as a potential contributing cause. Despite this combined approach, her pruritus remained a persistent, high-burden symptom for the remainder of her admission, fluctuating with her overall condition but never fully resolving.

**Table 2:** Liver biochemistry at the time of most severe pruritus

Parameter	Result	Reference range	Unit
Bilirubin	30	3-17	µmol/L
Alanine aminotransferase (ALT)	22	7-56	U/L
Alkaline phosphatase (ALP)	183	30-130	U/L
Gamma-glutamyl transferase (GGT)	Not measured	5-55 (female)	U/L
Albumin	31	35-50	g/L

She subsequently developed acute neurological deterioration, characterized by a new left facial droop, slurred speech, and reduced responsiveness, with a National Institutes of Health Stroke Scale score of 19. This was managed conservatively in keeping with her DNACPR status and her family's wishes. She transitioned to end-of-life care, and the CSCI was titrated upward to include morphine, midazolam, and levomepromazine. At her final review, she was comfortable and free of distress, with no further itch reported. She died on day 38 of her admission.

## Discussion

Pruritus in patients with advanced liver disease is often attributed to the underlying liver pathology, but in reality, the cause is usually multifactorial [1]. In our case, the focus was not on reaching a diagnosis but on relieving symptoms, as is often appropriate in the palliative setting. Several factors were considered, with chronic liver disease considered the most likely contributor to her pruritus. As outlined above, cholestatic disease carries a stronger association with hepatic pruritus than ARLD [7]. One large case-control study found that patients with chronic pruritus were nearly 70% more likely to have an ARLD diagnosis than controls (adjusted odds ratio, 1.69; 95% confidence interval, 1.43-1.98) [6]. Liver disease therefore remained a plausible contributor to her itch, even in the absence of a clear cholestatic biochemical picture.

Opioid-induced pruritus is a well-recognized, dose-related side effect, but it is easily overlooked when several symptoms are escalating and being treated at once [8]. This is thought to be a central effect of µ-opioid receptor activation rather than a true histamine-mediated allergic reaction [8]. Before her pruritus worsened, she was taking low-dose morphine as needed (2.5-5 mg), making opioid-induced pruritus unlikely. As her condition progressed, the CSCI morphine dose was increased to 10 mg and later to 20 mg during the period when her itch was most severe. The literature does not establish a specific dose threshold at which opioid-induced pruritus becomes likely. However, pruritus is recognized as a dose-related phenomenon, and the association with dose escalation in this patient is noted as an observation rather than evidence of causation [8]. Morphine was providing effective analgesia at the time, so the possibility that opioids were contributing to her itch does not appear to have been considered. Whether the increased dose played a role in worsening her pruritus cannot be confirmed.

Uremic pruritus is usually seen in patients with significant, often dialysis-dependent renal failure, not the mild-to-moderate impairment seen in this patient [4]. Pruritus related to chronic kidney disease (CKD) becomes substantially more prevalent at stages 4-5 (eGFR <30 mL/min/1.73 m²), with reported rates of 56% or higher, compared with a much lower prevalence at earlier stages [9]. Her eGFR of 49 corresponds to CKD stage 3a, so this was unlikely to have caused significant pruritus, though it may still have contributed alongside her other risk factors. Finally, large-volume paracentesis causes significant intravascular fluid shifts and can precipitate paracentesis-induced circulatory dysfunction [10]. A direct causal link between paracentesis and skin barrier disruption is not established in the literature, so this remains a hypothetical mechanism, raised here only as a possible contributor based on family-reported timing rather than confirmed pathophysiology. Her family felt this had also worsened her skin dryness following drain insertion, though this was not objectively measured. The large fluid shifts from paracentesis, combined with the sudden loss of abdominal wall tension, may disrupt the skin barrier and speed up water loss, potentially causing pre-existing hepatic pruritus to become more significant [11].

The management of hepatic pruritus follows a stepwise pattern. First-line agents include emollients and bile acid sequestrants such as cholestyramine, escalating to rifampin, opioid antagonists, and sertraline, which has been trialled for refractory symptoms [6]. In this case, "refractory" is used to describe the persistence of pruritus despite the treatments trialled; the full stepwise pharmacological ladder was not pursued given her palliative status, with most drugs requiring weeks to titrate, and this should be considered when interpreting the term. Antihistamines are also widely used in practice for hepatic pruritus [12]. There are two main molecular mechanisms of pruritus, and they can be divided into two primary neural pathways: histamine-dependent and histamine-independent pathways [12]. Acute itching due to an allergic reaction or insect bite relies heavily on histamine, whereas a chronic itch, as seen in our patient, is driven by a broad range of non-histaminergic mediators, which is consistent with this patient's incomplete response to fexofenadine [12]. Current guidance supports antihistamine use mainly for sedation rather than a targeted antipruritic effect in palliative care [2].

Levomepromazine is commonly used in the United Kingdom and European palliative care for nausea, agitation, and terminal restlessness [13]. It has broad receptor activity, including antihistaminergic effects, which may dampen the brain's processing of itch rather than treat a peripheral cause [13]. This gives it a recognized, though evidence-limited, role in refractory pruritus. Some subjective improvement followed its introduction, but it coincided with sedation, reinstatement of her emollient regimen, and progressive overall decline. This case therefore cannot be used to demonstrate the efficacy of levomepromazine as a specific antipruritic treatment.

This raises the question of whether her pruritus was truly refractory to medication, or whether something modifiable had simply been missed. Along with their other recommendations, dermatology suggested emollient therapy every two to three hours. Her family reported that the most noticeable improvement followed a large quantity of the emollient Epimax being applied every few hours, the same regimen used before admission. This was the first time clinically since admission that she appeared settled, comfortable, and asleep without visible scratching. There was no formal pruritus scoring and several treatments changed at once, so this cannot prove causation. In a hospital setting, reduced staffing ratios and less frequent one-on-one observation make it difficult to replicate the intensive emollient application she had received prior to admission. This is a limitation of ward-based care rather than a clinical oversight, and it highlights that holistic, non-pharmacological measures can be as important as escalating drug therapy [14]. Her progressively reducing consciousness may have limited her ability to perceive or communicate itch. Even allowing for this, the link with intensive emollient use suggests that restoring simple, familiar supportive care played a genuine and possibly leading role in her improvement.

## Conclusions

This case highlights the importance of a multidisciplinary approach to manage a complex case of multifactorial pruritus in palliative care. Dermatology reinstated her emollient regimen and confirmed there was no primary skin disease driving her symptoms, while palliative care repeatedly reinforced emollient use at every review, alongside their pharmacological input. Neither specialty acting alone would likely have reached the same conclusion. It also shows that simple supportive management can be overlooked and replaced with escalating pharmacological use. Continued escalation of medication carried real risks of sedation and polypharmacy in a frail patient. Regularly asking what is actually being treated, and why, can directly protect a patient's comfort at the end of life.
